# The effect of nano chitosan and calcium hydroxide intracanal medications on sealer penetration & bond strength in radicular dentin

**DOI:** 10.1186/s12903-026-08558-2

**Published:** 2026-05-21

**Authors:** Maha Nasr, Ahmed El-Banna, Maii Youssef Elmesellawy

**Affiliations:** 1 Egyptian Russian University, Cairo, Egypt; 2 Faculty of Dentistry, Ain Shams University, Cairo, Egypt; 3 Beni-Suef University, Beni-Suef, Egypt

**Keywords:** Sealer Penetration, Confocal Laser Microscopy, Push-out, Nano Chitosan

## Abstract

**Background:**

A three-dimensional obturation that provides a fluid tight seal of the root canal system is essential for long-term treatment success. Intracanal medicaments may hinder sealer penetration and/or affect its ability to bond with dentin. This study aimed to evaluate the sealer penetration and push-out bond strength after using Calcium hydroxide and a 2% nano-chitosan gel (CSNPs).

**Methodology:**

Twenty-four extracted single-canal lower premolars were randomly divided, according to the intra canal medicament used, into two groups: Group A, Ca(OH)_2_, and Group B, 2% CSNPs. After medication placement for 1 week, all canals were sealed with gutta-percha and AH Plus sealer, mixed with 0.1% fluorescent Rhodamine B isothiocyanate. Sealer penetration was evaluated with confocal laser microscopy and then push-out bond strength was measured for both groups.

**Results:**

For both groups, push-out bond strength varied significantly across root thirds (*p* < 0.001), with higher bond strength in the coronal third. The penetration radius values were significantly greater in the CSNPs group at all root levels (*p* ≤ 0.001). At the same time, no significant differences were found between groups or among root thirds in terms of penetration area ( *p* > 0.05).

**Conclusions:**

Both Ca(OH)_2_ and CSNPs showed comparable push-out bond strength along the root sections, with deeper, more uniform sealer penetration with CSNPs.

**Clinical relevance:**

Clinically, the choice between Ca(OH)_2_ and CSNPs should be guided by treatment objectives as both medicaments give comparable bond strength, while CSNPs offer advantages for deeper and more uniform tubular penetration.

## Introduction

The success of endodontic treatment depends on thoroughly cleaning the root canal system by removing pulp tissue, dentinal debris, and microorganisms. However, the complex canal anatomy renders it impossible for mechanical preparation alone to achieve complete eradication of intra-radicular infections [1, 2]. Thus, using chemical agents together with mechanical instrumentation in root canal treatment provides greater effectiveness in achieving the desired outcomes. In addition, for proper healing to occur, any residual antigens left in the canal after debridement must be inactivated. Consequently, this combined chemo-mechanical approach should be complemented with an appropriate intracanal medicament [3]. 

Even with meticulous preparation, irrigation, and placement of intracanal medications, complete eradication of endodontic pathogens is impossible because residual microorganisms are probably sheltered in areas that are difficult to access such as isthmuses, lateral and accessory canals, apical ramifications, deltas, and even inside the confines of the dentinal tubule, this makes entombment of those residual organisms of utmost importance, a function that clearly depends on how far the used root canal sealer can penetrate deep inside those dentinal tubules, how evenly the sealer is distributed and how effective it is in filling the discrepancies between the Gutta-percha core and the dentinal walls [4, 5]. 

Calcium hydroxide, widely used as an intracanal medicament, is recognized for its strong alkalinity and its capacity to destroy bacterial DNA [6]. Despite these advantages, Ca(OH)_2_ residues on root canal walls can reduce the penetration of sealers into the dentinal tubules and increase apical leakage by dissociating into calcium and hydroxyl ions over time [7]. Furthermore, the chemical reaction between these remnants and certain root canal sealers can negatively impact root canal sealing quality. Also, it can lead to decreased fluidity and working time [8].

Chitosan, a naturally derived polysaccharide obtained from crustacean shells, is safe, biocompatible, and fully biodegradable. It has been introduced to the endodontics field as a broad-spectrum antimicrobial material that also exhibits notable chelating properties [9]. Chitosan Nanoparticles (CSNPs) gel was successfully used in previous studies as an intracanal medicament [10]. An earlier study found that 2% CSNPs in gel form is as effective as calcium hydroxide against E. faecalis biofilm, with significantly less negative effects on dentine microhardness [11]. Limited evidence is available regarding the effect of CSNPs gel on sealer penetration and bond strength. Therefore, the current study aimed to compare the effects of 2% CSNPs with those of Ca(OH)_2_ on the penetration and bond strength of AH plus root canal sealer into dentinal tubules. The null hypothesis states that the impact of the two medications on sealer penetration or push-out bond strength wouldn’t be significantly different.

## Materials and methods

The manuscript of this laboratory study has been written according to Preferred Reporting Items for Laboratory studies in Endodontology (PRILE) 2021 guidelines.

### Sample size calculation

Based on a study by Cruz et al. [12], using G*Power (version 3.1.9.7), a sample size of 12 specimens per group (24 in total) was needed to identify a 20% difference in sealer penetration, based on a significance level of 0.05 and a study power of 0.8.

### Preparation and characterization of CSNPs

Chitosan nanoparticles (CSNPs) were synthesized using the ionotropic gelation method [13]. Briefly, chitosan (CS) at a concentration of 0.5% in 1% acetic acid was dissolved in distilled water at room temperature under magnetic stirring. Sodium tripolyphosphate (TPP), the cross-linking agent, was prepared separately at a concentration of 2 mg/mL. Using a 1 mL syringe, the TPP solution was added to the CS solution drop by drop, and the pH was subsequently adjusted to 4. Based on the CS concentration, the final CS-to-TPP mass ratio was maintained at 3:1. Unloaded nanoparticles were prepared by adding the TPP solution to the CS solution without any additional components. For characterization of the nanoparticles, their size and morphology were examined using transmission electron microscopy (TEM) with a JEOL JEM-2100 high-resolution instrument running at 200 kV accelerating voltage. TEM observations revealed the successful formation of uniformly spherical nanoparticles with a size distribution below 50 nm.

#### Preparation of the CSNPs gel

The synthesized CSNPs were resuspended in distilled water to obtain 5 mL of a 2% w/v solution, which was stirred for 30 min. Subsequently, 0.25 g of hydroxypropyl methylcellulose (HPMC; Loba Chemie, India) was gradually sprinkled into the solution at a mild temperature of 35 °C with vigorous stirring until a homogeneous gel was formed.

### Selection and preparation of samples

Twenty-four mandibular single rooted premolars, with complete root development and normal morphology extracted for orthodontic reasons were collected from the oral surgery department clinic, Beni-Suef University to be used for this study. Any defective, caries and/or cracked teeth were excluded. The current in vitro investigation methodology was authorized by the Ethics Committee (REC-FDBSU/07122023-10/NM). The teeth were disinfected by soaking in 5.25% sodium hypochlorite (NaOCl) for 15 min to remove any remaining tissue [14], then cleaned using an ultrasonic scaler to eliminate surface debris.

Following access cavity preparation, the working length for all samples was established by inserting a #15 K-file (Dentsply/Maillefer, Ballaigues, Switzerland) beyond the apex and subtracting 1 mm. Canals were prepared with ProTaper Universal rotary files (up to F4) (Dentsply/Maillefer, Ballaigues, Switzerland) using an endodontic motor (Endo-Mate TC2; NSK Nakanishi, Tochigi, Japan) at 300 RPM and 2 Ncm torque. Throughout instrumentation, canals were irrigated with 2.5% NaOCl( HCPC Clorox, Egypt) using gauge 30 side vented irrigation needles (Cerkamed, Poland).) Smear layer removal was done using NaOCl (5 mL of 2.5%), then 5 mL of 17% EDTA( PD, Switzerland) [11]. Samples were randomly divided into two groups (*n* = 12) using online software (https://www.ramdomizer.org): Group A received 2% nano chitosan gel (CSNPs), and Group B received calcium hydroxide (Ca(OH)_2_) ( UltraCal™, Ultradent). Medicaments were placed in the canals, excess was removed, and a moist cotton pellet was placed in the chamber before sealing with a temporary filling (Cavit™, 3 M ESPE, Germany). Teeth were stored at 37 °C and 100% humidity for one week.

**Fig. 1:**
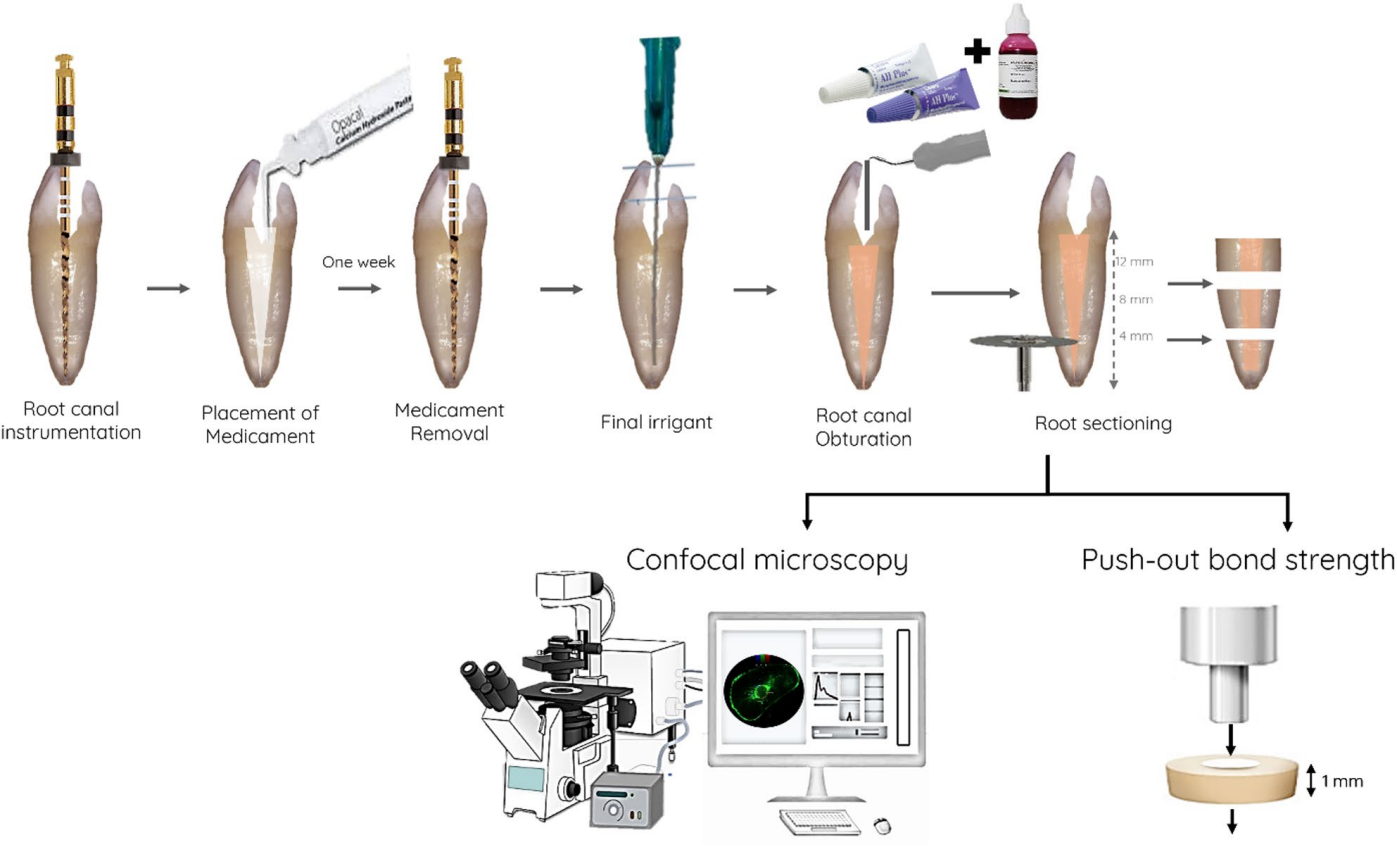
Schematic diagram representing steps for specimen preparation and testing

After a week, the temporary filling was removed, and the canals were irrigated with 1 mL of 2.5% NaOCl using a syringe fitted with a 30-gauge needle(Cerkamed, Poland). The canals were then cleaned to the working length using the master apical file (F4). Subsequently, all canals were flushed with 5 mL of 2.5% NaOCl followed by 5 mL of 17% EDTA, using the same type of needle for both groups. Obturation was performed using the warm vertical compaction technique (EQV, MetaBiomed, Korea) with AH Plus sealer (Dentsply Sirona, Germany), which had been mixed with 0.1% fluorescent Rhodamine B isothiocyanate (Merck, Darmstadt, Germany) for visualization. The quality of the obturation was confirmed radiographically. Access cavities were resealed, and all samples were stored at 37 °C and 100% humidity for one week to allow full setting (Fig. 1).

### Confocal laser microscopy

**Fig. 2:**
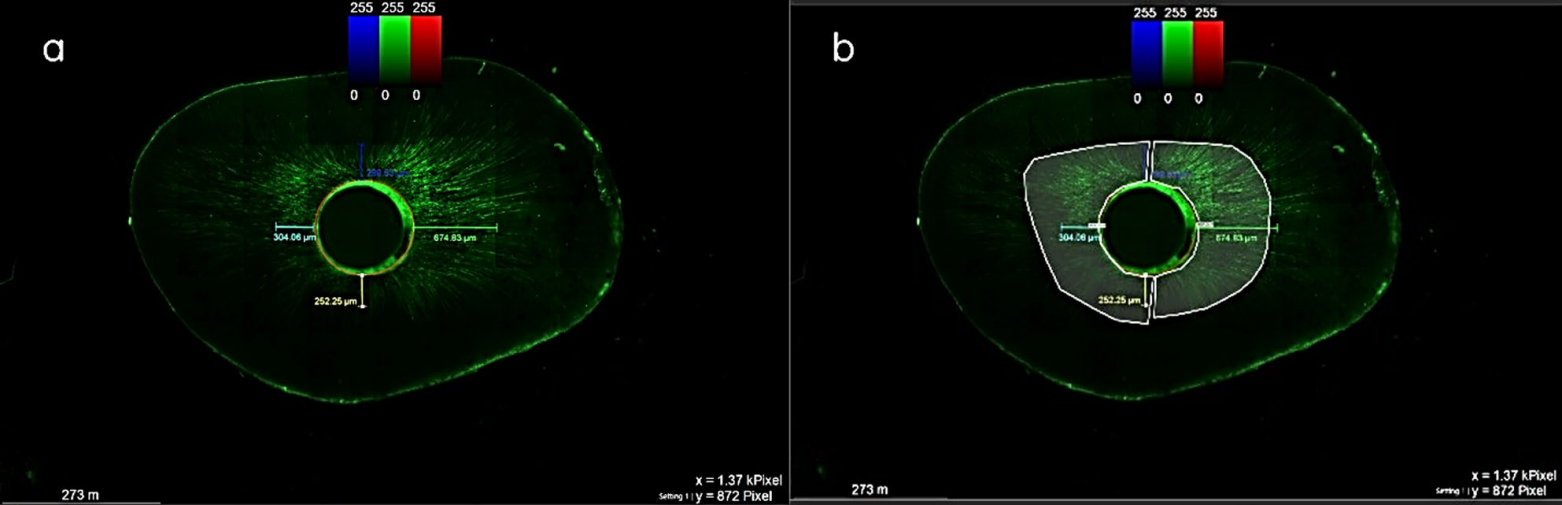
Representative Confocal microscopic images of dentinal tubular penetration of fluorescent-labelled root canal sealer, presenting (**a**) the maximum penetration depth, and (**b**) the penetration area

Roots were embedded and sectioned transversely at 4, 8, and 12 mm from the apex (apical, middle, and coronal, respectively) with a low-speed diamond disk mounted on an Isomet Precision diamond Saw (Isomet 4000, Buehler, Germany) under water cooling. Sections were then polished with silicon carbide abrasive paper, obtaining discs of 1 mm thickness. Images were acquired and analyzed using Leica Application Suite Leica DMi 8 Leica Stellaris 5 true confocal microscope upgrade set for Leica DMi 8 fluorescence microscope (Leica Microsystems, Germany) at a wavelength of 543 nm and 40X magnification after mounting onto glass slides. Subsequently, after acquiring the images, the following three parameters were calculated for each third (Fig. 2) [15].

1. *Maximum penetration depth*: This parameter was defined as the distance from the root canal wall to the deepest point of penetration at four standardized points with 90° angles. The total value of these four measurements was divided by 4 to calculate the mean maximum penetration depth (µm).
2. Penetration area: The extent of sealer penetration was quantified by measuring the total area of coverage, expressed in square micrometers (µm²) [16].

### Push out bond strength testing

Push-out bond strength was assessed using the same slices previously employed for sealer penetration evaluation. Stainless-steel plungers with diameters of 1.0, 0.5, and 0.35 mm were applied in the apical-to-coronal direction to load the filling material while avoiding contact with the canal walls. The test was performed using a universal testing machine (Instron 3365, Norwood, MA, USA), where the appropriate plunger was mounted on the upper compartment, and a cross-head speed of 1 mm/min was applied to load the root canal filling positioned on a supporting jig. Coronal and apical radii of the root slice were measured using a digital microscope (Dinolite, China) with the aid of Dinocapture 2.0 software. Push-out bond strength in MPa was calculated by dividing the maximum force (N) by the surface area of the root slice lumen (mm²). Adhesion area was calculated by using the following formula: A = π (R + r) h + π (R² + r²).

π = 3.14, h is the slice’s thickness, R is its coronal side radius, and r is its apical side radius [17]. 

## Statistical methods

Numerical data were presented as mean and standard deviation (SD)values. They were tested for normality and variance homogeneity by viewing the distribution and using Shapiro-Wilk’s and Levene’s tests, respectively. Both assumptions were valid for the push-out bond strength and radius data, and they were analyzed using two-way ANOVA. The assumptions were violated for the area data and were analyzed using two-way Aligned Rank Transform (ART) analysis. Comparisons of the main and simple effects of the estimated marginal means were made while using the error term of the main model and adjusting for multiple comparisons using the False Discovery Rate (FDR) method. The significance level was set at *p* < 0.05 within all tests. Statistical analysis was performed with R statistical analysis software version 4.5.0 for Windows.

## Results

For both Ca(OH)_2_ and CSNPs, the coronal third showed significantly higher push-out bond strength compared to the middle and apical thirds (*p* < 0.001). No statistically significant difference between the two medicaments was observed among the different root thirds (Table 1).

**Table 1:** Mean, SD, and P value for Push-out bond strength (MPa), sealer penetration Radius (µm), and Area (mm²^)^ in different root sections between the two groups

Measurement	Root third	Mean ± SD		p-value
		Ca(OH)_2_	CSNPs	
Push-out bond strength (MPa)	**Coronal**	16.65 ± 3.37^A^	17.00 ± 3.15^A^	**0.809**
	**Middle**	5.83 ± 1.20^B^	4.62 ± 1.07^B^	**0.057**
	**Apical**	3.81 ± 0.99^B^	4.06 ± 1.02^B^	**0.053**
	**p-value**	< 0.001*	< 0.001*	
Radius	**Coronal**	114.62 ± 25.47^A^	186.83 ± 38.41^A^	0.001*
	**Middle**	58.00 ± 13.31^B^	192.32 ± 45.16^A^	< 0.001*
	**Apical**	71.62 ± 12.57^AB^	213.83 ± 39.10^A^	< 0.001*
	**p-value**	0.024*	**0.378**	
Area	**Coronal**	32.63 ± 14.26^A^	42.23 ± 14.42^A^	**0.386**
	**Middle**	44.15 ± 15.22^A^	53.83 ± 18.17^A^	**0.699**
	**Apical**	81.44 ± 17.43^A^	72.50 ± 21.20^A^	**0.699**
	**p-value**	**0.156**	**0.240**	

Values with different superscripts within the same vertical column are significantly different

* significant (*p*<0.05)

The penetration radius values were significantly greater in the CSNPs group at all root levels (*p* ≤ 0.001), indicating superior dentinal tubule infiltration. Within the Ca(OH)_2_ group, the penetration radius varied significantly between regions (*p* = 0.024), with the coronal third showing the highest values. In contrast, the CSNPs group showed no significant intra-group differences (*p* = 0.378), suggesting more uniform and extensive tubule penetration.

Regarding penetration area, no statistically significant differences were found between groups or among root thirds (*p* > 0.05). Despite numerical variation, the large standard deviations suggest high variability and limited sensitivity of this measure in detecting intergroup differences.

## Discussion

Multidimensional sealing of the dentinal tubules encapsulates persistent bacterial load, leading to a substantial improvement in endodontic treatment, ensuring long-term success [18, 19]. While gutta-percha has been the standard core filling, sealers are essential to fill residual spaces and bond the core to canal walls, enhancing the hermetic seal and acting as a barrier against bacterial penetration and biofilm formation [19]. Sealer infiltration into dentinal tubules not only enhances the hermetic seal through mechanical interlocking with the dentin matrix, strengthening the bond, but also acts as a physical and chemical barrier that impedes bacterial penetration and biofilm formation, significantly reducing the risk of microbial persistence and reinfection [20]. Penetration depth is governed by smear layer removal, tubule diameter, sealer physicochemical properties, surface tension, and dentin contact angle [21].

Penetration depth and penetration area are commonly used parameters to assess sealer infiltration. Penetration depth indicates how far the sealer penetrates dentinal tubules, reflecting its ability to reach bacteria residing deep within. In contrast, the penetration area offers a more comprehensive evaluation by quantifying sealer coverage around the canal circumference, thereby reflecting both penetration depth and the uniformity of distribution. This makes the penetration area a more effective parameter for assessing sealer adaptation and its potential to prevent microleakage. Accordingly, our study measured both maximum penetration depth and penetration area [21]. 

Several methodologies have been employed to observe sealer penetration into dentinal tubules. These include scanning electron microscopy (SEM) [22, 23], light microscopy [24], and confocal laser scanning microscopy (CLSM) [25]. Among these, CLSM has recently gained prominence for its ability to effectively visualize sealer infiltration within subsurface structures, including dentinal tubules that are not exposed on the surface. It also distinguishes the depth and distribution of sealers within infected dentin. CLSM achieves this by optically sectioning specimens with focused laser beams to produce high-resolution, three-dimensional images. When combined with fluorescent dye as Rhodamine B, CLSM allows precise visualization and quantification of sealer penetration and does not alter sealer properties, as concentrations below 0.2% have been shown not to alter the physical characteristics of sealers [25–27]. Compared to SEM, CLSM offers clearer contrast between dentin and sealer penetration through a less sensitive technique, providing more precise information on sealer distribution [28].

Intracanal medicaments are essential for disinfection of necrotic teeth, [29]. Calcium hydroxide, being one of the most effective intracanal agents in endodontics, is regarded as highly effective in reducing residual microbial flora due to its potent alkaline nature, with a pH of approximately 12.5 [6]. Despite its broad-spectrum antibacterial properties, calcium hydroxide failed to totally eliminate bacteria from infected root canals. It can be challenging to remove from the root canals completely, often leaving residues that may interfere with sealer penetration and adhesion to dentin [7]. Additionally, its high alkalinity presents other disadvantages, such as reducing dentine microhardness [30].

Antibacterial nanoformulations have demonstrated significant potential in enhancing drug delivery in endodontics, enabling sustained release, and improving treatment efficacy. The use of nanoparticles also supports a more sustainable and efficient approach by reducing drug dosage, minimizing toxicity risks, and optimizing material use [31]. Nano-chitosan, a promising irrigating solution [32–34] and intracanal medicament exhibits promising hydrophilic properties, bioactivity, biodegradable action, biocompatible nature, and broad-spectrum antimicrobial properties, especially against E. faecalis, the famous resistant bacterial strain in persistent endodontic infections [11, 35, 36], and Candida albicans, based on electrostatic interaction, which leads to cell membrane disruption [37].

The present study demonstrates that while Ca(OH)_2_ and CSNPs provide comparable overall canal filling based on cross-sectional area measurements, their effect on sealer penetration into dentinal tubules varies significantly by material and root region. The push-out bond strength varied significantly across root thirds for both Ca(OH)_2_ and (CSNPs) groups. In the coronal third, Ca(OH)_2_ and CSNPs exhibit the highest bond strength, with no significant difference between them. Penetration is typically higher in the coronal region because it is more accessible to instrumentation and irrigation during chemo-mechanical disinfection, matching the known anatomical pattern of wider dentinal tubules with greater density that enhances sealer adhesion [15, 38].

CSNPs showed greater radius measurements in most areas, meaning they allowed the sealer to spread further into the tubules, relatively uniform across all root thirds, with no significant differences, indicating more consistent dentinal tubule penetration or adaptation among CSNPs regardless of root level. CSNPs’ nanoscale particle size, along with their superior rheological properties and diffusibility, allows them to navigate and infiltrate the narrow and complex dentinal tubule structure more effectively than Ca(OH)_2_ [39].

In contrast, Ca(OH)_2_’s larger particle size may limit its ability to penetrate deeply, particularly in the apical third with the narrower tubules. On the other hand, the coronal tubules are larger and more numerous than the apical ones, allowing greater Ca(OH)_2_ penetration in the coronal region. The flowability of sealers is essential for proper adaptation to the walls and penetration into dentinal tubules. In the current study, the used sealer was AH plus, an epoxy resin-based sealer which is known to properly wet both dentin and gutta-percha; however, Ca(OH)_2_ exposure was reported to significantly reduce dentin wettability [40]. It was also reported that it changed the intracanal pH and maintained an alkaline environment for up to 14 days [41]. While epoxy resin sealers have a neutral or acidic pH [42]. Those findings may explain why the depth of sealer penetration in the Ca(OH)_2_ group was significantly lower than in the CSNPs group. It also affects the characteristics of the sealers.

No significant differences in penetration area were found between groups or root thirds. This can be attributed to the high variability (as shown by large standard deviations) and the lower sensitivity of area as a measurement parameter. Unlike depth or radius, penetration area can be more prone to distortion from canal irregularities, making it less reliable for detecting subtle differences in sealer performance [43]. Tubule morphology and variability affect measurements like penetration area and suggests that such metrics may be limited in comparative analysis unless sample standardization is strictly maintained. The lack of significant differences in cross-sectional area proves that both materials fill the canal space equally well.

Clinically, choosing between Ca(OH)_2_ and CSNPs should be guided by treatment goals: Ca(OH)_2_ may be preferable when apical bond strength and seal integrity are priorities. At the same time, CSNPs offer advantages for deeper, more uniform tubule penetration and disinfection [44]. Keeping in mind that both medicaments had the greatest bond strength coronally, with no significant difference between them, a quality that is most important in resisting coronal microleakage, as a common cause for endodontic treatment failure [45, 46]. Considering root anatomy and material-specific properties is essential for optimizing long-term treatment outcomes.

A limitation of the present investigation is the potential difference in the sealer penetration and bond strength under in vitro versus in vivo conditions due to variability of the dye penetration and specimen preparation. The in vitro testing eliminates possible effects of the dynamic biological environment of the oral cavity, including temperature fluctuations and occlusal loads, which could alter the performance of both the root canal medicament and the root canal sealers.

## Conclusions

Within the limitations of the current study, it could be concluded that 2% of the Chitosan Nanoparticles provide a superior depth of resin sealer penetration compared to Calcium hydroxide with a similar penetration area.

For clinical relevance, 2% of CSNPs represent a viable option as a naturally derived intracanal medication with comparable results to the most commonly used medicament, Ca(OH)_2_. From a clinical perspective, the choice between Ca(OH)₂ and CSNPs should depend on the treatment objectives. CSNPs offer advantages for deeper, more uniform tubular penetration.

### Recommendations

Long-term clinical studies to evaluate the endodontic treatment outcome after using CSNPs or Ca(OH)_2_ intracanal medications are advised to correlate the findings of this investigation with the clinical reality.

## Data Availability

The authors confirm that the data supporting the findings of this study are available within the manuscript and its supplementary materials.
